# Juggling Between Parental and School Expectations: The Development of Domain‐Specific Acculturation Orientations in Early Adolescence

**DOI:** 10.1111/jora.12547

**Published:** 2020-01-29

**Authors:** Jana Vietze, Maja K. Schachner, Linda Juang, Fons J.R. van de Vijver, Peter Noack

**Affiliations:** ^1^ University of Potsdam; ^2^ Erasmus University Rotterdam; ^3^ College for Interdisciplinary Educational Research (CIDER); ^4^ Tilburg University; ^5^ North‐West University; ^6^ University of Queensland; ^7^ Higher School of Economics; ^8^ Friedrich Schiller University Jena

## Abstract

We examined how perceived acculturation expectations from parents and school, and ethnic discrimination predicted early adolescents’ heritage and mainstream acculturation orientations at home (private domain) and in school (public domain) one year later. We surveyed 263 early adolescents of immigrant background in Germany (*M_age_* = 10.44 years, 60% female). Multigroup path analyses revealed that perceived acculturation expectations and ethnic discrimination were more strongly related to adolescents’ private than public acculturation orientations. Parental heritage expectations were the strongest predictor of adolescents’ acculturation orientations. Boys were more susceptible than girls to ethnic discrimination and acculturation expectations in school, which affected their private and public acculturation orientations. Results highlight the importance of integrating domain‐specific and gendered experiences when analyzing adolescents’ acculturative development.

Adolescents face challenges, such as becoming increasingly independent from parents, broadening their peer networks, and developing a coherent sense of social and cultural self (Eccles, Lord, & Roeser, 1996; Umaña‐Taylor et al., 2014). In addition, adolescents of immigrant background1In the current study, we use the terms “of immigrant background” and “ethnic minority” to refer to ethnic minority populations in Europe and the United States. In Germany, as in most of continental Europe following the Holocaust, issues of ethnicity and race have been mainly discussed regarding immigrants versus cultural mainstream or having versus not having an immigrant background (Motti‐Stefanidi & Masten, 2013). In line with the German legal term, we use the term “of immigrant background” to refer to anyone who has at least one parent who did not have citizenship of the country of settlement (e.g., Germany) at birth (Göttsche, 2017). In common vernacular, the term has become racialized and is often used to refer to visible minorities of color, particularly those of Turkish, Arab, or African descent (Elrick & Schwartzman, 2015). Despite specific experiences of ethnic minority groups (Bornstein, 2017), children and youth of disadvantaged minorities often share heightened perceptions of (ethnic) discrimination and the negative consequences for individual well‐being (Schmitt, Branscombe, Postmes, & Garcia, 2014). Thus, the literature review in this study is largely based on research on disadvantaged ethnic minority samples, including samples of immigrant background, from Europe and the United States. meet acculturative challenges at home, in school, and in society (Motti‐Stefanidi, Berry, Chryssochoou, Sam, & Phinney, 2012), including the perceived support or pressure for cultural group membership (Phinney, Horenczyk, Liebkind, & Vedder, 2001). When spending time at home (private life domain) or in school (public life domain), adolescents may differ in their acculturation orientations (Güngör & Bornstein, 2009), meaning how adolescents emphasize their heritage, ethnic culture, and the mainstream culture in which they live (Arends‐Tóth & van de Vijver, 2006; Tadmor & Tetlock, 2006). A strong orientation toward both cultures is related to favorable individual adjustment outcomes (Nguyen & Benet‐Martínez, 2013) and is partly predicted by expectations which individuals perceive in different life domains about how they should acculturate (i.e., perceived acculturation expectations; Kunst & Sam, 2013). As early adolescents spend increasing amounts of time outside the family home and with peers in school (Brown & Larson, 2009), they may be confronted with discordant or concordant acculturation expectations in these two domains. However, domain specificity of perceived acculturation expectations and acculturation orientations has been mostly tested with adult populations (Arends‐Tóth & van de Vijver, 2006; Noels & Clément, 2015).

In the current study, we focus on early adolescents of immigrant background in Germany and how their perceptions of acculturation expectations at home and in school relate to their acculturation orientations at home and in school over the first year of secondary school. To our knowledge, this is the first study to investigate longitudinal associations between acculturation expectations and acculturation orientations within and across life domains and in early adolescence, known as a sensitive period characterized by high instability of the social self (Meeus, 2011).

Perceived ethnic discrimination is not only a well‐established risk factor for psychological maladjustment (Benner et al., 2018), but also an important predictor of adolescents’ changes in cultural orientations (Lepshokova, Lebedeva, & van de Vijver, 2017). Furthermore, early adolescence is a critical period for gender‐role development (Ruble, Martin, & Berenbaum, 2007), and adolescent girls and boys vary in their acculturative experience (Suárez‐Orozco & Qin, 2006), and in their perception of societal pressures, such as personal and group discrimination (Güngör & Bornstein, 2009). In our previous cross‐sectional study, mean differences suggested that early adolescent boys were already more at risk than girls of developing a negative acculturation trajectory by feeling more discriminated against, separating from the mainstream society, and engaging in delinquent behavior at school (Schachner, van de Vijver, & Noack, 2018). By adding longitudinal associations and domain‐specific acculturation outcomes, this follow‐up study allows us to test whether boys decrease in their mainstream orientation in school as a result of higher perceived ethnic discrimination by mainstream society compared with girls (Güngör & Bornstein, 2009). We thus contribute to previous research on domain specificity by adding perceived ethnic discrimination as predictor, and by exploring gender variation longitudinally and as a moderator of perceived domain‐specific acculturation expectations and acculturation orientations of early adolescents.

## Acculturation in Early Adolescence

Acculturation refers to the changes in cultural practices, values, and identities, and their influence on individuals’ psychological well‐being and social functioning, when people of different cultures interact for an extended time (Berry, 2003; Ward, 2001). The widely used bidimensional approach (e.g., Berry, 2003) suggests that both orientations toward the heritage and mainstream culture can be conceptually combined within four different acculturation strategies: integration (i.e., both orientations high), assimilation (i.e., high mainstream and low heritage orientation), separation (i.e., low mainstream and high heritage orientation), and marginalization (i.e., both orientations low). The integration strategy is regarded as most beneficial for youth adjustment outcomes (see Nguyen & Benet‐Martínez, 2013, for a meta‐analysis). However, classifying orientations into all four strategies, also statistically, has been difficult to replicate and criticized as not strictly comparable across studies (van de Vijver, 2017; Ward & Geeraert, 2016). Thus, in this study we consider heritage and mainstream orientation as two conceptually independent dimensions of acculturation orientation, as is common with this bidimensional approach (e.g., Ouarasse & van de Vijver, 2005; Rudmin, 2003).

Adolescence is a developmental period for negotiating different aspects of the social self (Erikson, 1968), and for forming acculturation orientations (Berry, Phinney, Sam, & Vedder, 2006). In multicultural environments, studies have mostly targeted changes in ethnic minorities’ heritage and mainstream orientation from mid‐adolescence to late adolescence (Güngör & Bornstein, 2009; Schwartz et al., 2013). Early adolescence is characterized by a low stability of the sense of self (see Meeus, 2011, for a review), and developmental milestones, such as the transition from primary to secondary school, demanding re‐adaptation under changing social conditions (Eccles et al., 1996). However, early adolescence has been understudied regarding acculturation orientations and the process of forming a cultural sense of self. Therefore, the current study analyzes acculturative changes in the first year of secondary school, as a sensitive period in which adolescents move from early toward mid‐adolescence.

## Acculturation Orientations in the Private and Public Life Domain

Acculturation orientations are likely to vary between life domains (Birman, Simon, Chan, & Tran, 2014; Miller et al., 2013). Cross‐cultural psychologists have distinguished between acculturation orientations in the more personal, social–emotional private life domain (e.g., family or religious community), and the more visible, functional public life domain (e.g., school or workplace; Arends‐Tóth & van de Vijver, 2006). Findings across immigrant generations, age, and ethnic groups show that adolescents and adults emphasize the heritage culture mainly in the private life domain and the mainstream culture in the public or both life domains (Güngör & Bornstein, 2009; Navas, Rojas, Garcia, & Pumares, 2007; Noels & Clément, 2015).

Within life domains, more specifically, acculturation orientations may differ between behavioral domains (e.g., language, social interactions, daily habits) and values domains (e.g., belief systems, world views, political ideologies; Miller, 2010). Acculturation in behavioral domains, in particular, has been related to ethnic minorities’ adjustment outcomes, including acculturative stress, well‐being, and occupational adjustment (Birman et al., 2014; Miller, 2010). However, to our knowledge, there are no studies investigating whether already in early adolescence behavioral aspects of acculturation differ between life domains, and how this depends on antecedents at home, in school, and in society. Our study aims to fill this gap.

## Acculturation Expectations by Parents and in School, and Ethnic Discrimination

Ethnic minority youth develop a cultural sense of self based on the support and guidance of heritage and mainstream group members (Gartner, Kiang, & Supple, 2014; Kiang & Fuligni, 2009; Motti‐Stefanidi et al., 2012; Sam & Oppedal, 2003; Wang & Benner, 2016). Not only adolescents’ acculturation orientations can differ between life domains but also the expectations adolescents perceive about how they should acculturate at home, in school, and in mainstream society (Kunst & Sam, 2013; Navas et al., 2007; Tip et al., 2015).

At home, ethnic minority parents are often, but not invariably, more oriented to the heritage culture than their children (Fuligni, 2012). Many studies have focused on parents’ ethnic–racial socialization which deals with parental practices of transmitting values, beliefs, and behaviors of their ethnic or racial group or groups to their children (Hughes et al., 2006). In contrast, parental acculturation expectations refer to the parents’ preference to what extent their child should endorse the heritage culture (i.e., parent heritage expectations) and the mainstream culture (i.e., parent mainstream expectations), including customs and traditions, language use, and contact with peers from the heritage or mainstream culture (Schachner, van de Vijver, & Noack, 2014). At home, adolescents are likely to perceive high expectations about maintaining their families’ heritage culture, though parents also hold expectations to adopt the mainstream culture (Rasmi & Costigan, 2018). Early adolescents’ own acculturation preferences are likely to conform to perceived parental acculturation expectations, as well as perceived expectations by peers of immigrant background: If adolescents perceive that parents or peers prefer them to endorse the heritage culture and not the mainstream culture, adolescents may indeed show high separation tendencies, and a high heritage and low mainstream orientation (Kunst & Sam, 2013; Schachner et al., 2014).

In contrast, school is the primary context for interactions with mainstream culture peers and teachers (Horenczyk & Tatar, 2012). Across European countries, youth perceive and encounter strong preferences by mainstream members to adopt the mainstream culture than to maintain the heritage culture (Groenewold, de Valk, & van Ginneken, 2014; Kunst & Sam, 2013). Questionnaire studies in Germany and Chile showed that perceiving high expectations in school for contact with mainstream culture peers (i.e., school mainstream expectations) may relate to adolescents’ strong mainstream orientations (Zagefka & Brown, 2002; Zagefka, González, & Brown, 2011).

Perceptions of ethnic discrimination are likely a covariate for the link between adolescents’ perceived acculturation expectations and acculturation orientations. Latent profile analysis has recently linked perceiving ethnic discrimination to parents’ ethnic–racial socialization over time (Kiang, Supple, & Stein, 2018). The authors argued that perceiving high amounts of ethnic–racial socialization at home may increase adolescents’ awareness of cultural issues outside of home, including a higher perception of ethnic discrimination. In addition, ethnic discrimination by mainstream teachers is part of ethnicity‐related school experiences for many students with immigrant background in Germany (Moffitt, Juang, & Syed, 2018).

Furthermore, youth experiencing personal and group discrimination may result in a lower identification with the mainstream culture in adolescence and adulthood (Fleischmann & Verkuyten, 2016; Musso, Inguglia, & Coco, 2015), a mechanism commonly referred to as rejection‐disidentification (for a review, see Verkuyten & Martinovic, 2012). In addition, mainstream societies in many European countries, including Germany, hold strong assimilation preferences for immigrants and their descendants (Jasinskaja‐Lahti, Liebkind, Horenczyk, & Schmitz, 2003; López‐Rodríguez, Zagefka, Navas, & Cuadrado, 2014; Zick, Wagner, Van Dick, & Petzel, 2001). In Germany, youth who experience mainstream members’ prejudice against ethnic minorities may result in a stronger orientation toward the heritage than mainstream culture (Branscombe, Schmitt, & Harvey, 1999), referred to as rejection‐identification (Christ, Asbrock, Dhont, Pettigrew, & Wagner, 2015). However, rejection‐identification findings have been less consistent than rejection‐disidentification findings among immigrant populations across Europe (Bobowik, Martinovic, Basabe, Barsties, & Wachter, 2017). In this study, we investigate whether greater perceived ethnic discrimination relates to a lower mainstream and higher heritage orientation among early adolescents of immigrant background.

In the current study, to understand adolescents’ acculturative and developmental changes, we follow recent claims to further contextualize the environment on the family, institutional, and societal level (van de Vijver, 2017; Ward & Geeraert, 2016). Together with the notion of domain specificity, we expected associations between perceived acculturation expectations and ethnic discrimination with early adolescents’ acculturation orientations to differ between life domains (Hypothesis 1). More precisely, we expected early adolescents’ heritage orientation to be stronger at home (private life domain) than in school (public life domain), and mainstream orientation to be stronger in school than at home at Time 1 (H1a). We anticipated that higher perceived parental heritage expectations at Time 1 would promote a stronger heritage orientation and weaker mainstream orientation at Time 2, whereas higher perceived school mainstream expectations would promote a stronger mainstream orientation. We further expected more perceived ethnic discrimination at Time 1 to promote a lower mainstream and higher heritage orientation at Time 2 (H1b). Finally, we tested whether perceived parental acculturation expectations at Time 1 relate more strongly to adolescents’ acculturation orientations at home (private life domain) than in school (public life domain) at Time 2, and perceived school acculturation expectations and ethnic discrimination relate more strongly to adolescents’ acculturation orientations in school than at home (H1c).

## Gender Differences in Longitudinal Associations

Scholars have repeatedly emphasized the importance of gender when studying acculturation (Güngör & Bornstein, 2013; Schwartz & Montgomery, 2002; Suárez‐Orozco & Qin, 2006). Across various North American and Western European countries, life domains, and periods of adolescence, girls have shown higher levels of mainstream orientation and fewer difficulties in engaging with the mainstream society than boys (Berry et al., 2006; Güngör & Bornstein, 2009). A possible reason is that boys report higher and increasing perceptions of personal and group‐based ethnic discrimination over the course of adolescence compared to girls (Güngör & Bornstein, 2009), and that a large amount of ethnic discrimination happens in the public life domain, including by school personnel, peers, or societal institutions (Benner & Graham, 2013). As a result, boys also show lower levels of sociocultural adjustment (e.g., in dealing with authorities or mainstream culture members) in predominantly mainstream contexts such as school or society (Güngör & Bornstein, 2009; Schachner et al., 2018).

In Germany, early adolescent girls and boys perceive comparable levels of mainstream and heritage expectations at home and in school (Schachner et al., 2018). However, being more susceptible to ethnic discrimination than girls, boys may learn to rely more on support at home, and increasingly use rejection‐disidentification as a coping mechanism at home and in school (Güngör & Bornstein, 2013). In contrast, girls seem to rely less on mainstream expectations for their acculturation outside of home (Güngör & Bornstein, 2009). Girls often face higher academic expectations by teachers than boys (De Boer, Bosker, & van der Werf, 2010). Girls also perceive mainstream language brokering for their families as less stressful (Buriel, Love, & De Ment, 2006) and report a higher mainstream language proficiency in late adolescence compared with boys (Güngör & Bornstein, 2013). Thus, even when experiencing ethnic discrimination, girls may be more encouraged to maintain a stable mainstream orientation over time and across life domains than boys.

In the current study, we expected gender variations in associations between acculturation conditions at Time 1 and acculturation orientations at Time 2 (Hypothesis 2). More specifically, we expected boys to be more affected by school mainstream expectations and ethnic discrimination at Time 1 regarding acculturation orientations at home and in school at Time 2 compared with girls (H2a). In contrast, for girls and boys and across both life domains, we expected a similar mechanism that links parental acculturation expectations at Time 1 and early adolescents’ acculturation orientations at Time 2 (H2b).

## Methods

### Participants and Procedure

This study included self‐reports of 263 early adolescents of immigrant background in Germany (at first assessment: *M_age_* = 10.44 years, *SD_age_* = .62, range_age_ = 9–12 years, 60% female). Most participants (90%) were of the second immigrant generation, meaning that participants themselves were born in Germany, with both parents born outside of Germany. For most participants (84%), parents originated from the same country. Participants represented the largest ethnic minority groups in Germany, such as the Turkish‐ (38%), Italian‐ (10%), Russian‐, Kosovar‐, Greek‐, and Bosnian‐heritage (11% each). Participants attended either the university‐preparatory high academic school track (39%), the medium vocational track (38%), or the low vocational track (23%). Regarding language fluency, most participants (89%) reported a very good or good fluency in German, and 72% reported a very good or good fluency in their heritage language.

Participants were surveyed during class time and in German, at the beginning of secondary school (5^th^ grade, Time 1) and one year later (6^th^ grade, Time 2). This study was part of a larger research project on acculturation and intercultural relations involving students with and without immigrant background in 22 culturally diverse secondary schools in southwestern Germany (Schachner et al., 2018). To assure comparability across schools and school tracks, we selected classrooms with a similar share of students with immigrant background. This meant that some academic track schools in this study had an above‐average share of immigrant students compared with other academic track schools. Participation was based on the permission from school authorities and at least one parent’s active consent. During the instruction immediately prior to data collection, a trained researcher reminded students that participation was voluntary and could be interrupted or stopped any time. The study’s ethical standards were approved by the Ministry of Education of the federal state of Baden‐Württemberg in Germany.

### Measures

For measures not originally available in German, we employed a translation back‐translation method (van de Vijver & Leung, 1997). Existing measures were adapted based on qualitative interviews with 14 students of immigrant background by making these more comprehensible for our adolescent age group (e.g., by simplifying the language), and by tailoring the contents to the German context. For example, in line with common vernacular in Germany at the time of the first data collection (2010), we replaced references to ethnic or racial minorities with “foreigners,” and references to participants’ or their families’ ethnic or cultural heritage with “from my other country.” A trained researcher explained this terminology to participants immediately before data collection. A pilot study with 51 early adolescents of immigrant background provided reliability and validation information for all items and scales prior to the study. Scale reliabilities are presented in Table 1.

**Table 1:** Descriptive Statistics, Reliabilities, and Bivariate Correlations Among Study Variables at Time 1 and Time 2

	1	2	3	4	5	6	7	8	9	10	11	12	13	14	15	16	17
*Control variables T1*																	
1 age	‐	−.10	−.02	−.02	.03	.08	−.10	.04	.05	−.03	.10	.02	.03	−.02	−.02	.10	−.14
2 SES^a^	.10	‐	−.01	−.04	−.02	−.06	.12	.02	.03	−.06	.01	−.11	.07	.05	.10	−.17	.10
3 Classroom proportion of students of immigrant background	−.11	.01	‐	−.17*	.12	−.12	.02	.15	.05	.02	−.36***	.12	−.11	.04	−.25*	.21*	−.19*
4 School type	−.11	−.02	−.20*	‐	−.01	.05	−.22**	−.01	−.04	−.12	.00	−.04	.14	−.13	.08	−.01	.37**
Acculturation conditions T1																	
5 Parent heritage expectations	−.02	.03	.06	.01	‐	.47***	.20*	.11	.24***	.28***	−.02	.43***	.13	.32**	−.19	.23*	.01
6 Parent mainstream expectations	−.03	.00	−.04	−.11	.39***	‐	.09	.11	−.19*	.03	.34***	.05	.52***	.18	.03	−.04	.16
7 School heritage expectations	−.12	.05	.17	−.18	.33***	.25*	‐	−.07	−.02	.26***	.18*	.07	−.01	.04	.07	−.05	−.04
8 School mainstream expectations	−.02	.07	.12	.00	.33***	.27**	.10	‐	.25***	.02	−.17*	.10	.15	−.10	−.11	.06	−.04
9 Perceived discrimination	.02	−.04	.15	.00	.19*	−.15	.03	.18	‐	.15	−.17*	.33***	−.19*	.10	−.02	.35**	−.02
*Acculturation orientations T1*																	
10 Heritage orientation at home	−.02	−.01	−.04	.18	.43***	.17	.21*	.16	.05	‐	−.04	.21**	−.04	.38**	.04	.25**	.03
11 Mainstream orientation at home	−.02	.17	−.15	.02	−.03	.51***	.16	.19	−.30***	.20*	‐	−.11	.25***	.11	.36**	−.12	.14
12 Heritage orientation in school	−.09	.02	.10	.01	.62***	.16	.14	.14	.31***	.24*	−.13	‐	−.03	.14	−.18	.43**	−.11
13 Mainstream orientation in school	−.07	.05	−.04	.03	.16	.59***	.13	.12	−.24*	.08	.59***	−.08	‐	.04	.12	−.11	.29**
*Acculturation orientations T2*																	
14 Heritage orientation at home	−.07	−.12	.07	.08	.36**	−.06	.15	.25*	.13	.33**	−.15	.37**	−.03	‐	−.08	.45**	−.04
15 Mainstream orientation at home	−.01	−.04	−.18	−.03	−.17	.31**	−.04	−.14	−.41**	−.21	.35**	−.12	.48**	−.26*	‐	−.17	.35**
16 Heritage orientation in school	−.07	−.09	.06	−.04	.34**	.14	.24*	.14	.21	.19	.00	.59**	.00	.36**	−.20	‐	−.07
17 Mainstream orientation in school	−.02	−.11	−.20	−.06	.07	.34**	−.01	−.05	−.35**	.04	.38**	−.14	.54**	−.11	.49**	−.08	‐
*M (SD)* girls	10.47 (.68)	−0.36 (.92)	0.69 (.17)	‐	4.03 (.85)	3.63 (.95)	3.10 (.97)	3.09 (1.03)	1.86 (.73)	3.96 (.98)	3.17 (1.12)	3.40 (1.16)	4.12 (.90)	4.01 (.07)	3.16 (1.16)	3.71 (1.15)	4.16 (0.89)
*M (SD)* boys	10.39 (.60)	−0.53 (.85)	0.71 (.18)	‐	3.98 (.96)	3.57 (1.06)	3.28 (1.03)	3.02 (1.03)	2.34 (1.05)	3.70 (1.06)	3.04 (1.15)	3.41 (1.26)	3.82 (1.12)	4.03 (.94)	2.80 (1.19)	3.82 (1.11)	3.63 (1.19)
*α/ r*_SB_ girls	‐	‐	‐	‐	.79	.83	.71	.70	.70	.68^b^	.53^b^	.70^b^	.56^b^	.68^b^	.62^b^	.80^b^	.60^b^
*α/ r*_SB_ boys	‐	‐	‐	‐	.89	.88	.77	.72	.85	.68^b^	.66^b^	.75^b^	.69^b^	.60^b^	.62^b^	.78^b^	.76^b^

Notes

*N* = 158 girls and *N* = 105 boys. Correlations among girls above and correlations among boys below the diagonal.

a Socioeconomic status, combined score for family economic capital and educational background.

b Spearman‐Brown coefficient for scales with two items.

* *p* < .05, **p < .01, ***p < .001.

#### Perceived acculturation expectations

This scale was adapted from a Dutch scale for adults (Arends‐Tóth & Van de Vijver, 2007) to measure adolescents’ perceptions of acculturation expectations by parents and in school, regarding cultural customs and traditions, language use, and contact with peers from the heritage or mainstream culture (Schachner et al., 2014). The scale consisted of 12 items for perceived parental acculturation expectations, with six items concerning *parental heritage expectations* (e.g., “My parents want me to get to know the customs and traditions from my other country,” “My parents want me to have a good command over the language of my other country,” or “My parents want me to have friends from my other country”), and six items concerning *parental mainstream expectations* (mirrored from parental heritage expectation; e.g., “My parents want me to get to know the customs and traditions from Germany”). The scale further included eight items for perceived school acculturation expectations, with four items measuring *school heritage expectations*, including two items for expectations from mainstream peers and two items for expectations from teachers (e.g., “The German children in my class think that it’s fine when foreign children behave in school as is typical in their other country,” or “The teachers in my class think that it’s fine when foreign children in school also go by what is customary in their other country”). The four other items measured *school mainstream expectations*, again including two items each for mainstream peers and teachers (mirrored from school heritage expectations; e.g., “The German children in my class think that foreign children should behave in school like German children”). Participants responded on a 5‐point Likert scale from (1) no, that’s not right to (5) yes, that’s right.

#### Perceived ethnic discrimination

We used the general measure for perceived ethnic discrimination from the ICSEY survey (Berry et al., 2006), addressing the perceived frequency of being treated unfairly or negatively due to one’s cultural heritage. The measure included a total of five items tapping into personal discrimination (e.g., “Have you ever been teased or insulted because you are from your other country?”) as well as group discrimination (e.g., “Have you ever experienced that people treated other people from your other country unfairly or poorly?”). Responses ranged from (1) never to (5) very often.

#### Domain‐specific acculturation orientations

We adapted the original 18‐item scale from a Dutch measure for adults (Arends‐Tóth & Van de Vijver, 2007). As with perceived acculturation expectations, we selected eight items that comprised participants’ acculturation orientations at home (private life domain) and in school (public life domain). The scale tapped into behavioral domains of acculturation (Miller et al., 2013), including individual preferences for customs and traditions, language use, and contact with heritage and mainstream culture members. It consisted of four subscales: For the private life domain, the scale included two items for *heritage orientation at home* (“I like the way families live in my other country” and “I like how parents from my other country treat their children”), and two items for *mainstream orientation at home* (mirrored from heritage orientation at home; e.g., “I like the way families live in Germany”). For the public life domain, the scale included two items for *heritage orientation in school* (“In school, I like speaking the language of my other country” and “In school, I like spending time with children from my other country”), and two items for *mainstream orientation in school* (mirrored from heritage orientation in school; e.g., “In school, I like speaking German”). The response scale ranged from (1) no, that’s not right to (5) yes, that’s right. To test reliabilities, we used the Spearman‐Brown coefficient, which is the recommended measure for two‐item scales (Eisinga, Te Grotenhuis, & Pelzer, 2013).

#### Covariates

As important covariate, we added participants’ age because gender differences in acculturation orientations are likely to increase over the course of adolescence (e.g., Güngör & Bornstein, 2009). We also included socioeconomic status (SES), because it is confounded with ethnic minority and migration status and therefore with experiences of acculturation and discrimination (Vedder, Sam, & Liebkind, 2007). To assess SES, we combined well‐established indicators of economic capital and family educational background into a single factor (e.g., Schachner, Brenick, Noack, van de Vijver, & Heizmann, 2015). For economic capital, we used the 3‐item Family Affluence Scale (FAS; Boyce, Torsheim, Currie, & Zambon, 2006; German version by Richter & Leppin, 2007), including the number of cars in the household—(0) none, (1) one, or (2) two or more; whether the child has his or her own room—(0) no or (1) yes; and how many times the family has been on holiday during the past year—(0) not at all, (1) once, (2) twice, or (3) three times or more. For family educational background, we assessed the number of books in the household on a 5‐point Likert scale from (1) none or very few to (5) more than 200 books (e.g., Bos et al., 2007).

We further included important school‐level covariates of acculturation (Schachner, Juang, Moffitt, & van de Vijver, 2018), namely the classroom proportion of students of immigrant background and school type. We estimated the classroom proportion of students of immigrant background for each participant, based on information provided by the schools (*M*_prop_ = .70; range_prop_ = .18–.94). Scores closer to 0 indicated a low percentage, whereas scores closer to 1 indicated a high percentage of students of immigrant background in the classroom. For school type, in southwestern Germany, parents choose their children’s secondary school track, often in line with teacher recommendations based on students’ academic performance in primary school. However, teacher recommendations can be biased, and students of immigrant background are less likely to be recommended to academic track schools than their mainstream peers, even with similar performance (Glock, Krolak‐Schwerdt, & Pit‐ten Cate, 2015).

### Plan of Analyses

First, we examined mean level differences in study variables at Time 1 and Time 2. To examine early adolescents’ domain‐specific acculturation orientations, we conducted a series of repeated‐measures MANOVAs with heritage and mainstream orientation at home and in school at the beginning of secondary school (Time 1) and one year later (Time 2). Next, to test for gender mean differences at Time 1, we conducted a MANCOVA with acculturation orientations at home and in school, and perceived acculturation expectations by parents and school as dependent variables. Age, socioeconomic status (SES), school type, and classroom proportion of students of immigrant background were treated as covariates. We previously established gender mean differences in ethnic discrimination in the same sample, where early adolescent girls reported a lower perceived ethnic discrimination at Time 1 than boys (Schachner et al., 2018).

Second, to analyze gendered associations between acculturation conditions at Time 1 and acculturation orientations at Time 2, we performed longitudinal multigroup path analyses in *Mplus 7.3* (Muthén & Muthén, 1998‐2011), with gender as a grouping variable. We used full information maximum‐likelihood estimation for missing data (total of 13% for girls, and 12% for boys)*.* Participants were nested in their classrooms (*n* = 40) because perceived acculturation expectations in school may depend on unobserved factors at the classroom level (e.g., varying levels of teaching about cultural diversity or school climate). Due to the complexity of the models and our specific focus on domain‐specific acculturation orientations, analyses were conducted separately for acculturation orientations at home (private acculturation model) and for acculturation orientations in school (public acculturation model) as dependent variables at Time 2. In both models, the dependent variables were predicted by perceived parental and school acculturation expectations and ethnic discrimination at Time 1, and controlled for age, SES, and acculturation orientations at Time 1 to account for stability over time.

We started with building an unconstrained private acculturation model, which was followed by a model with all regression paths set to be equal between groups (*structural weights*). Then, using the model constraint option in *Mplus*, we individually identified and released regression paths that differed significantly between girls and boys. Next, we constrained all correlations between predictors to be equal across groups (*structural covariances*). We repeated the same steps with the public acculturation model. We assessed model fit using the comparative fit index (CFI) and the root mean squared error of approximation (RMSEA), with CFIs greater than .95 and RMSEAs <.06 indicating a good model fit (Hu & Bentler, 1999). We compared different models with the ∆CFI, which compared to other conventional measures (e.g., ∆χ^2^) is much less affected by sample size (Cheung & Rensvold, 2002). A change in CFI of no more than .01 indicated support for the more restricted model.

## Results

In the following sections, we first report preliminary analyses of study variables, followed by repeated‐measures MANOVA and MANCOVA results about mean differences at home and in school at Time 1 (H1a), and between girls and boys. Next, we introduce the two multigroup longitudinal regression models that explored associations between perceived acculturation conditions at Time 1 and domain‐specific acculturation orientations at Time 2 (H1b, H1c, H2).

### Preliminary Analyses, Domain‐Specific, and Gendered Mean Differences

Descriptive statistics and bivariate correlations between study variables at the beginning of secondary school (Time 1) and one year later (Time 2) are presented in Table 1. Bivariate correlations between acculturation orientations at home and in school were weak to moderate (between *r* = .21 and .25 for girls; between *r* = .24 and .59 for boys) and justified separate analyses for both life domains. For girls and boys, perceived parental heritage and mainstream expectations were moderately correlated. When participants perceived high parental mainstream expectations, they also perceived high school mainstream expectations at Time 1. Similarly, when they perceived high parental heritage expectations, they also perceived high school heritage expectations, but also high ethnic discrimination from society.

In line with findings from mid‐adolescence and late adolescence (Güngör & Bornstein, 2009), the repeated‐measures MANOVA revealed intra‐personal, domain‐specific mean differences of heritage and mainstream orientations at Time 1 (H1a). There were large (Cohen, 1988) multivariate main effects for acculturation orientations at home, both at Time 1, *F*(1, 262) = 65.41, *p* < .001, (partial) η^2^ = .20, and at Time 2, *F*(1, 178) = 69.17, *p* < .001, η^2^ = .28, and for acculturation orientations in school at Time 1, *F*(1, 261) = 36.83, *p* < .001, η^2^ = .12, but not at Time 2, *F*(1, 177) = 2.67, *p =* .10, η^2^ = .02. Thus, at home participants endorsed the heritage culture more than the mainstream culture at both time points, whereas in school participants endorsed the mainstream language and culture more than the heritage language and culture, but only at Time 1. Upon closer inspection, there was a significant, yet small interaction effect of acculturation orientations in school × gender at Time 2, *F*(1, 175) = 7.74, *p* = .01, η^2^ = .04. Thus, in school at Time 2, only girls reported a higher mainstream than heritage orientation, *F*(1, 103) = 10.57, *p* = .002, η^2^ = .09, but not boys, *F*(1, 73) = 1.00, *p* = .32, η^2^ = .01.

MANCOVA results indicated a significant multivariate main effect for gender at Time 2, *F*(4, 159) = 2.68, *p =* .03, η^2^ = .06, but not at Time 1, *F*(8, 234) = 1.62, *p =* .12, η^2^ = .05. At Time 2, univariate effects were small to medium, *F*(1, 162) = 10.44, *p* = .001, η^2^ = .06, with girls reporting a higher mainstream orientation in school than boys, but not at home.

Furthermore, we found a significant multivariate main effect for classroom proportion of students of immigrant background at Time 1, *F*(8, 234) = 3.60, *p* = .001, η^2^ = .11. The medium univariate effect indicated that participants in classrooms with more students of immigrant background reported a lower mainstream orientation at home, *F*(1, 241) = 18.17, *p* < .001, η^2^ = .07, and higher perceived school expectations for students of immigrant background to adopt the mainstream culture, *F*(1, 241) = 7.05, *p* = .01, η^2^ = .03, compared to participants in classrooms with fewer students of immigrant background. There were no significant multivariate effects for age, socioeconomic status, or school type.

### Testing Hypotheses Using Longitudinal Multigroup Regression Analyses

The main goal of this study was to explore gender differences in direct associations between perceived acculturation conditions at Time 1 and acculturation orientations at Time 2 in the private and public life domain. Table 2 displays fit statistics for all multigroup longitudinal regression models, from unconstrained to structural covariance. For the private acculturation model (regarding adolescents’ acculturation orientations at home), the structural covariance model with three parameters freed was accepted as the most restrictive model with a good fit (χ^2^/*df* = 0.97, *p* = .56, RMSEA = .00 [90% CI from .00 to .05], CFI = 1.00). This means that girls and boys showed significant differences in their regression paths from school mainstream expectations to heritage orientation at home, from ethnic discrimination to mainstream orientation at home, and in the correlation between school mainstream expectations and mainstream orientation at home at Time 1. For the public acculturation model (regarding adolescents’ orientations in school), we accepted the structural covariance model with two parameters freed as the most restrictive model with a good fit (*χ* ^2^/*df* = .95, *p* = .61, RMSEA = .00 [CI from .00 to .04], CFI = 1.00). Therefore, girls and boys differed in their associations between school heritage expectations and heritage orientation in school, and between ethnic discrimination and mainstream orientation in school. In the private and public acculturation models, girls and boys showed a low stability of acculturation orientations at home and a medium stability of acculturation orientations in school over time. Table 3 displays the unstandardized and standardized results of the private and public multigroup regression models.

**Table 2:** Fit Statistics for Multigroup Regression Analyses

	χ^2^/*df*	RMSEA	CFI	ΔCFI
*Private acculturation model*				
M1: Unconstrained	1.04	0.34	0.99	‐
M2: Structural Weights	1.97	0.08	0.78	0.21
M3: M2 and PME → HO released	1.97	0.08	0.8	0.19
M4: M3 and SME → HO released	1.68	0.07	0.87	0.12
M5: M4 and ED → MO released	1.26	0.04	0.96	0.03
M6: M5 and structural covariances	1.09	0.03	0.93	0.06
*M7: M6 and SME with MO released*	*0.93*	*0*	*1*	*<0.01*
*Public acculturation model*				
M8: Unconstrained	0.8	0	1	‐
M9: Structural Weights	1.54	0.06	0.89	0.11
M10: M9 and SHE → HO released	1.31	0.04	0.95	0.05
M11: M10 and ED → MO released	1.05	0	1	0.00
*M12: M11 and structural covariances*	*0.86*	*0*	*1*	*0.00*

Note

Most restrictive model with a good fit in italics. PME = parental mainstream expectations, HO = adolescent heritage orientation, SME = school mainstream expectations, ED = ethnic discrimination, MO = adolescent mainstream orientation, SHE = school heritage expectations. Model fit as is compared with unconstrained model (ΔCFI).

**Table 3:** Results of the Structural Weights Multigroup Regression Models

	Private acculturation model						Public acculturation model					
	Adolescent heritage orientation at home (T2)			Adolescent mainstream orientation at home (T2)			Adolescents heritage orientation in school (T2)			Adolescent mainstream orientation in school (T2)		
*R* ^2^		.22/ .25			.15/ .26			.26/ .34			.20/ .22	

Control variables (T1)	*b*	*(SE)*	β	*b*	*(SE)*	β	*b*	*(SE)*	β	*b*	*(SE)*	β
Age	−.10	(.10)	−.05/−.05	−.07	(.14)	−.03/−.03	.02	(.10)	.01/.01	−.16	(.11)	−.15/−.15
SES	.04	(.07)	.03/.03	.02	(.09)	.01/.01	−.13	(.07)	−.13/−.13	−.01	(.06)	−.01/−.01
Classroom proportion of students of migrant background	.16	(.42)	.12/.13	−.72	(.51)	−.08/−.09	.25	(.46)	.03/.03	−.71	(.44)	−.13/−.12
School type	−.06	(.07)	−.04/−.04	.00	(.11)	.01/.01	.00	(.08)	.01/.01	.17	(.10)	.14/.14
Adolescent heritage orientation at home	.28**	(.07)	.29/.30		‐			‐			‐	
Adolescent mainstream orientation at home		‐		.24*	(.10)	.23/.21		‐			‐	
Adolescent heritage orientation in school		‐			‐		.44***	(.07)	.46/.48		‐	
Adolescent mainstream orientation in school		‐			‐			‐		.33***	(.08)	.36/.34

Acculturation conditions (T1)	*b*	*(SE)*	β	*b*	*(SE)*	β	*b*	*(SE)*	β	*b*	*(SE)*	β
Parent heritage expectations	.31**	(.10)	.30/.30	−.27**	(.09)	−.20/−.22	−.04	(.12)	−.03/−.03	.01	(.09)	.01/.02
Parent mainstream expectations	−.05	(.07)	−.05/−.05	.18	(.10)	.16/.16	−.01	(.09)	−.01/−.01	.02	(.09)	.03/.03
School heritage expectations	−.08	(.07)	−.09/−.09	.02	(.09)	.02/.02	−**.06/.22** *	**(.12/.09)**	−**.06/.21**	−.02	(.06)	−.06/−.06
School mainstream expectations	−**.16/.10**	**(.09/.08)**	−**.18/.09**	−.10	(.08)	−.10/−.09	.05	(.09)	.06/.06	−.07	(.07)	−.09/−.09
Perceived ethnic discrimination	.03	(.07)	.03/.03	**.19/**−**.25** *	**(.14/.11)**	**.13/**−**.22**	.13	(.10)	.13/.13	**.06/**−**.23**	**(.09/.14)**	**.05/**−**.21**

Notes

Predictors at Time 1 and acculturation orientations at home (private acculturation model) and in school (public acculturation model) at Time 2. Coefficients of girls on the left, boys on the right of the dash. Parameters freed to vary between girls and boys in bold.

* *p* < .05, **p < .01, ***p < .001.

We found partial support for our first set of hypotheses regarding domain specificity and relative changes in early adolescent acculturation orientations depending on perceived acculturation conditions at Time 1. We confirmed our findings on domain‐specific mean differences (H1a) in that parental heritage expectations at Time 1 were associated with participants’ stronger heritage orientation and weaker mainstream orientation at home at Time 2 (H1b). However, against predictions, there were no significant effects of school mainstream expectations on participants’ mainstream orientations. Partly as expected, ethnic discrimination was related to adolescents’ lower mainstream orientation one year later, but only reached significance at home and not in school, and only for boys and not for girls. Ethnic discrimination was also not related to a higher heritage orientation. In line with expectations, we found large (Adachi & Willoughby, 2015) standardized effects for parental acculturation expectations predicting participants’ acculturation orientations at home, and for school acculturation expectations predicting participants’ (but only boys’) acculturation orientations in school (H1c).

In line with our second set of hypotheses about gender variation, girls and boys differed in their associations between perceived public acculturation conditions (school acculturation expectations, ethnic discrimination) at Time 1, and acculturation orientations at Time 2. We did not find the expected longitudinal stronger associations between school mainstream expectations and acculturation orientations for boys compared to girls (H2a). However, as an additional finding and only for boys, perceiving high school heritage expectation at Time 1 was associated with an increase in heritage orientation in school over the first year of secondary school. As expected, when boys perceived high ethnic discrimination, their mainstream orientation decreased over the year, but this medium effect only reached significance for mainstream orientation at home and not in school. In contrast, girls showed opposite, yet non‐significant effects between ethnic discrimination and mainstream orientation at home and in school. As expected, girls and boys did not vary in their associations between perceived parental acculturation expectations at Time 1 and acculturation orientations at Time 2 in both domains (H2b).

## Discussion

In this study of girls and boys of immigrant background in Germany, we investigated their changes in acculturation orientations within and between the private and public life domain over the first year of secondary school. We analyzed whether early adolescents’ perceptions of parental acculturation expectations, school acculturation expectations, and ethnic discrimination would predict participants’ acculturation orientations at home (private life domain) and in school (public life domain) one year later. Our results partially confirmed that associations differed between perceived acculturation conditions and acculturation orientations when comparing the private and public life domains (Hypothesis 1). We further found gender variations in means and associations between perceived public acculturation conditions (school acculturation expectations, ethnic discrimination) in adolescents’ first year at secondary school and acculturation orientations one year later (Hypothesis 2). We first discuss the most important findings regarding life domain and gender variations. We conclude with limitations and implications for future research.

In line with previous research on older populations, early adolescents showed a higher orientation toward the heritage than mainstream culture at home, in the private life domain (Arends‐Tóth & van de Vijver, 2006; Güngör & Bornstein, 2009; Noels & Clément, 2015). However, in this study, one year into secondary school only girls and not boys reported a higher orientation toward the mainstream than heritage culture in school, a more visible public life domain. Our findings support that boys of immigrant background might be less inclined to adopt the mainstream culture than girls and have more difficulties adjusting in the predominantly mainstream context of school (e.g., Berry et al., 2006). Gender differences may become increasingly apparent throughout adolescence (Güngör & Bornstein, 2009). Not identifying with the mainstream culture may limit boys’ future participation in public life, including the educational or job context, and intercultural contact in society (Arends‐Tóth & van de Vijver, 2006). As mainstream culture values and behaviors are inherent in school curricula and school acculturation expectations (Zagefka et al., 2011), schools may hold a key role in providing access to the mainstream culture throughout adolescence.

Early adolescents acculturated in line with perceived acculturation expectations from parents and school (Schachner et al., 2014; Zagefka et al., 2011). Longitudinal associations showed the expected domain‐specific link: Perceived parental expectations predicted early adolescents’ acculturation orientations at home one year later, and perceived school expectations predicted acculturation orientations in school, but the latter was only found for boys. Participants’ acculturation orientations were less stable at home than in school. A possible explanation is that individuals with and without immigrant background have similar preferences for how minorities should acculturate in the public life domain but conflicting preferences for the private life domain (Navas et al., 2007). Furthermore, perceived parental expectations to maintain the heritage culture were the strongest predictor of participants’ acculturation orientations. Early adolescents may seek most cultural guidance with their parents as the main context for the heritage culture, and may only focus on activities outside of home when moving toward mid‐adolescence (Brown & Larson, 2009). This highlights again the importance of family relations for immigrant families and for adolescents’ cultural orientation (Rumbaut, 2005), especially at the transition to secondary school.

Boys who perceived high levels of ethnic discrimination in their first year in secondary school reported a lower mainstream orientation at home one year later. This confirms previous findings from mid‐adolescence and older adolescents in Europe that boys may not only perceive more personal and group discrimination than girls, but they may also be more inclined to use rejection‐disidentification at home as a coping mechanism (Güngör & Bornstein, 2013; Musso et al., 2015). One possible explanation is that ethnic minority adolescents often have greater exposure to the mainstream cultural norms and values than their parents, and may become ambassadors for the mainstream culture at home (Fuligni, 2012). As levels of perceived ethnic discrimination increase over the course of secondary school (Greene, Way, & Pahl, 2006; Umaña‐Taylor, 2016), boys may withdraw from the mainstream culture not only in school, but may also feel less need for establishing mainstream culture elements at home.

Another possible explanation can be found in studies on biculturalism, meaning individuals who simultaneously endorse two or more cultures (Schwartz, Birman, Benet‐Martínez, & Unger, 2016). Bicultural individuals may be disadvantaged with regard to school engagement and school performance when facing discrimination (Baysu, Phalet, & Brown, 2011). The more boys feel discriminated against, especially by teachers (Moffitt et al., 2018), the more they may perceive that heritage and mainstream cultures are incompatible, and the more they may endorse either culture, but not both (Lepshokova et al., 2017). As a result, highly stigmatized individuals of immigrant background are at risk to turn away from the mainstream society in several life domains, and may attribute a lack of personal success and societal engagement to cultural barriers in society (Azghari, van de Vijver, & Hooghiemstra, 2018). Here, social workers in school or other adults of immigrant background may serve as role models for boys in constructing a positive and cohesive sense of cultural self in an adverse societal climate.

If boys perceived high acceptance in school for heritage culture maintenance in their first year in secondary school, they were more strongly oriented toward the heritage culture in school one year later (Zagefka et al., 2011). However, we did not find that boys were also more sensitive to mainstream expectations at home or in school than girls (Güngör & Bornstein, 2009). Still, our finding gives a positive outlook that schools can play a significant role in facilitating a diversity climate that fosters not only the mainstream, but also heritage culture orientation (Schachner, Noack, van de Vijver, & Eckstein, ; Schachner et al., 2018). School is the primary context for interactions with mainstream peers and teachers (Horenczyk & Tatar, 2012). Thus, a school that is supportive of students’ heritage culture may hold strong potential to encourage boys’ cultural orientation, especially when boys perceive heightened discrimination.

### Limitations and Future Research

This study also has some limitations. First, the current study was restricted to two time points, leading to the assumption of linear changes in variables from Time 1 to Time 2. For a more accurate description of changes, more time intervals would be needed. Second, almost half of the students in our study were of Turkish descent, representing the largest ethnic minority group in Germany with particular acculturation experiences (Vietze, Juang, Schachner, & Werneck, 2018). We are aware of the restricted generalizability of results, and acculturation‐related experiences are likely to differ by ethnic minority group or heritage country (Diehl, Lubbers, Mühlau, & Platt, 2016; Vedder et al., 2007). Third, our study investigated the impact of perceived acculturation expectations on acculturation orientations but could not reveal whether expectations were perceived as positive and supportive or as negative and threatening. Furthermore, our adolescent‐reported measures could not detect a match or mis‐match between actual and perceived acculturation expectations, which may be confounded with characteristics of family relationships, such as parent–adolescent conflict or an intergenerational acculturation gap (Fuligni, 2012). Thus, multi‐informant measures should be introduced in future studies. Fourth, although this was common terminology at the time of data collection, we are aware that using the terms “Germans” and “foreigners” in our questionnaire may have reinforced a perspective of a white in‐group and perceived immigrant other (Moffitt & Juang, 2019). We encourage future research to use more accurate and inclusive terms that represent and acknowledge the heterogeneity of the population of interest. Finally, we were interested in gender‐specific experiences but did not explicitly measure these. In Germany, different religious groups hold similar gender‐role expectations regarding the private life domain (e.g., household chores; Becher & El‐Menouar, 2014). However, gender‐role expectations may depend strongly on aspects such as traditionalism or religiosity (Diehl, Koenig, & Ruckdeschel, 2009), which need to be incorporated in future research.

## Conclusion

Despite the limitations, the current study is novel in two ways. First, it is one of the first longitudinal studies to combine the notions of domain‐specific and gendered acculturation orientations among early adolescent girls and boys. This study supports that domain specificity is a highly important aspect of acculturation, providing a more holistic understanding of basic acculturation processes (Arends‐Tóth & van de Vijver, 2006; Miller et al., 2013). Yet, different developmental contexts hold different demands depending on gender or age. Second, to our knowledge, it is the first study considering changes in early adolescent acculturation orientations, depending on perceived acculturation expectations at home and in school, and ethnic discrimination by mainstream society. This is important because adolescents’ acculturation and development take place in a variety of social contexts, which are all interconnected (Motti‐Stefanidi et al., 2012). We conclude that early adolescent boys may be more susceptible than girls to perceived acculturation conditions in the public life domain (i.e., school acculturation expectations, ethnic discrimination) and how they relate to acculturative processes. Finally, we emphasize that further knowledge is needed on gender differences in the perception of supportive acculturation conditions to generate a better understanding for acculturation as a dynamic, domain‐specific, and gendered phenomenon, and to help shape supportive environments for adolescent development.
